# Detection accuracy for epithelial dysplasia using an objective autofluorescence visualization method based on the luminance ratio

**DOI:** 10.1038/ijos.2017.37

**Published:** 2017-11-10

**Authors:** Nanami Yamamoto, Koji Kawaguchi, Hisako Fujihara, Mitsuhiko Hasebe, Yuta Kishi, Masaaki Yasukawa, Kenichi Kumagai, Yoshiki Hamada

**Affiliations:** 1 Department of Oral and Maxillofacial Surgery, School of Dental Medicine, Tsurumi University, Yokohama, Japan; 2 Department of Oral Hygiene, Tsurumi Junior College, Yokohama, Japan

**Keywords:** autofluorescence, epithelial dysplasia, oral cancer, Visually Enhanced Lesion scope

## Abstract

The autofluorescence visualization method (AVM) uses blue excitation light to assist in the diagnosis of epithelial dysplasia. It detects epithelial dysplasia as a black area, which is known as fluorescence visualization loss (FVL). In this study, we evaluated the detection accuracy for epithelial dysplasia of the tongue using the objective AVM and assessed its possible clinical utility. Seventy-nine tongue specimens clinically suspected to have leukoplakia or squamous cell carcinoma (SCC) were analyzed. First, the AVM was subjectively performed using the Visually Enhanced Lesion scope (VELscope), and the iodine-staining method was then performed. After biopsy, the histopathological results and the luminance ratio between the lesion and healthy tissue were compared, and a receiver operating characteristic curve was created. The cutoff value for the objective AVM was determined; the lesion was considered FVL-positive or -negative when the luminance ratio was higher or lower than the cutoff value, respectively. The histopathological diagnoses among the 79 specimens were SCC (*n*=30), leukoplakia with dysplasia (*n*=34), and leukoplakia without dysplasia (*n*=15). The cutoff value of the luminance ratio was 1.62, resulting in 66 FVL-positive and 13 FVL-negative specimens. The luminance ratio was significantly higher in the epithelial dysplasia-positive than -negative group (*P*<0.000 1). The objective AVM showed much higher consistency between histopathological results than did the two methods (kappa statistic=0.656). In conclusion, objective autofluorescence visualization has a potential as an auxiliary method for diagnosis of epithelial dysplasia.

## Introduction

Epithelial dysplasia is often observed in the tissue surrounding oral squamous cell carcinoma (SCC),^1^ and it is reportedly associated with a malignant transformation rate of 2.2%–38.1%.^2, 3^ Therefore, residual epithelial dysplasia after surgical treatment of oral SCC is an important risk factor for a poor prognosis,^4, 5^ and precise detection of affected areas with epithelial dysplasia prior to surgical resection of oral SCC is important to prevent local recurrence. The iodine-staining method (IOM) has been used to detect oral SCC and epithelial dysplasia.^1, 6, 7^ In the IOM, healthy oral mucosa turns to a yellowish brown color secondary to the iodine-glycogen reaction; however, epithelial dysplasia of the oral mucosa, which has less glycogen, is visually detected as an iodine-unstained area because of the absence of this reaction. Although the IOM is a useful modality with which to detect epithelial dysplasia, it has several weak points: iodine is irritative to the mucosa, it is contraindicated in patients with allergy to iodine,^7^ and it cannot be used in keratinized mucosa, such as the gingiva and hard palate, because of the mechanism of iodine staining.^1^

The autofluorescence visualization method (AVM) is another modality that has a potential for detecting epithelial dysplasia without the above-mentioned weak points of the IOM.^8^ The AVM utilizes blue excitation light to detect the difference in luminance between healthy tissue and epithelial dysplasia. No drugs or other agents are required.^7, 9, 10, 11, 12^ Recently, several optical imaging system have been produced such as Visually Enhanced Lesion scope (VELscope, White Rock, BC, Canada), EVINC (MMOptics, São Carlos, Brazil), and Identifi 3000 (DentalEZ Inc, Malvern, PA, USA), using different wavelength of light sources.^11, 13, 14^

**Figure 1:**
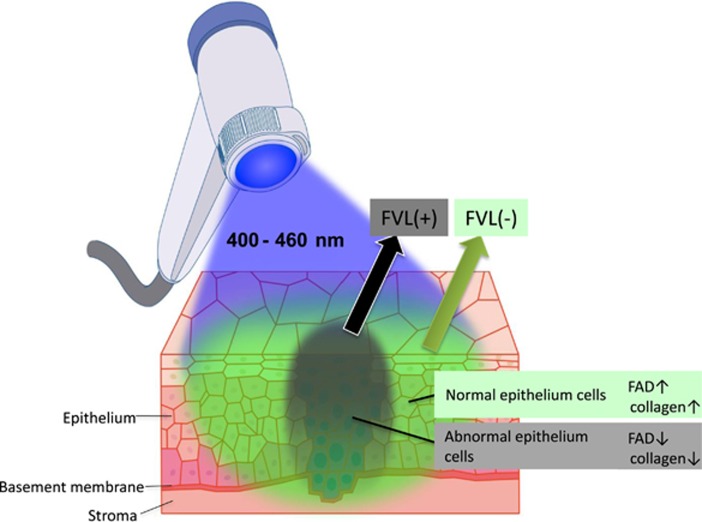
**Mechanism of autofluorescence visualization method using the VELscope.** Blue excitation light from the VELscope is emitted to the oral mucosa, and the fluorescence energy from the endogenous autofluorescence substances in the healthy mucosa emits green light. Conversely, low levels of endogenous autofluorescence substances in the tissue affected by epithelial dysplasia cannot emit green light. Therefore, epithelial dysplasia can be detected as fuorescence visualization loss (FVL), which is shown as a dark area.

The AVM detects epithelial dysplasia by the following mechanism. Healthy oral mucosa contains abundant endogenous autofluorescence substances such as collagen and flavin adenine dinucleotide. When blue excitation light (wavelength of 400–460 nm) emitted from the VELscope contacts these endogenous autofluorescence substances, the level of fluorescence energy emitted from the endogenous autofluorescence substances will become lower than that of the light energy emitted from the VELscope.^15, 16^ Green light with a wavelength of 515 nm is then emitted from endogenous autofluorescence substances in healthy area.^17, 18^ However, the collagen cross-links and basal lamina of tissue affected by SCC with epithelial dysplasia are destroyed,^19, 20^ and glucose is highly consumed in malignant tissue even when in an aerobic environment (Warburg effect).^21^ The concentration of flavin adenine dinucleotide, which is the main source of cellular fluorescence, then decreases in the epithelial dysplasia.^22, 23, 24^ Therefore, the lower level of endogenous autofluorescence substances in tissue affected by epithelial dysplasia and SCC of the oral mucosa has no fluorescence energy, resulting in the appearance of a black area detected as fluorescence visualization loss (FVL) (Figure 1).

In previous studies, the AVM had been performed to detect FVL by visual inspection (subjective AVM) resulting in relatively large variability in the diagnostic accuracy; for example, sensitivity varied from 30.0% to 100.0%, and specificity varied from 15.3% to 80.8%.^9, 10, 12, 25, 26, 27^ The sensitivity and specificity of EVINCE also resulted in this range, however, the result showed significant variation between unskilled examiner and adept examiner.^14^ Therefore, to elucidate these variability possibly based on surgeon’s skill or wonder for judgment, we tried to quantify the luminance of FVL and normal tissue (lesion-to-normal tissue uptake ratio; LNR) by image processing software as the objective AVM in this study. We also evaluated the possible clinical indications for the objective AVM in comparison with the subjective AVM and the conventional IOM.

## Materials and methods

### Patients

We examined 79 specimens obtained from 62 patients (31 male and 31 female) with an average age of 59.6 years (mean, 60.5 years; range, 21–85 years) who were referred to our department and underwent biopsy under a clinical diagnosis of leukoplakia or SCC of the tongue from February 2014 to November 2016 (Table 1). This study was approved by the Institutional Ethical Committee of Tsurumi University School of Dental Medicine on 12 February 2014 (No. 1139), and all participants signed an informed consent agreement.

### Summary of protocol

The first step of the procedure was a conventional oral examination including palpation. The subjective AVM was carried out for the area visually suspected to be affected by epithelial dysplasia and the presence of FVL was illuminally detected subjectively. Next, a photograph was taken for the subjective AVM. Then, the iodine-unstained area was detected by the IOM. These procedures were performed by two specialists in oral and maxillofacial surgery who had 19 and 27 years of clinical experience in oral cancer treatment. The luminance ratio between the lesion and healthy tissue was then calculated using image-analysis-software. Consequently, biopsy was performed and the result of the histopathological analysis was compared with the luminance ratio, and the cutoff value for the objective AVM was determined. Then, the objective AVM was assessed on the basis of this semi-quantitative value. The detection accuracy of each modality (the objective AVM, the subjective AVM and the conventional IOM) was also analyzed.

### Imaging apparatus and photographing conditions

All photographs of lesion sites were taken by single-lens reflex camera (EOS Kiss X5; Canon, Tokyo, Japan) equipped with an 18- to 55-mm macro lens (Canon) and macro ring light (MR-14 EX; Canon). The photographing conditions were as follows: the ISO film speed was AUTO, shutter speed was 1/125 s, and *F*-value was 8.0. Photos at visual inspection and the IOM were taken with a flash in a room with white light, and those for the AVM were taken without a flash and with the room light turned off.

### Subjective AVM

The subjective AVM was performed using a VELscope and VELscope camera attachment kit (doctorseyes LLC, Vista, CA, USA). The distance between the emission site of the VELscope and lesion site was set to 15 cm, and photographs were taken under blue excitation light. The green light area was defined as healthy mucosa area, and the black area of FVL was defined as lesion area.

### IOM protocol

First, the target lesion on the tongue was washed with physiological saline solution and air-dried, and 1.5% diluted iodine was then applied. After 3 min, the area was fully rinsed and all surplus iodine liquid was removed. Consequently, the areas stained and unstained by iodine were confirmed. The stained area was defined as the epithelial dysplasia-negative area, and the unstained area was defined as the epithelial dysplasia-positive area.

### Histopathological diagnosis

For biopsy, the lesion was resected including both the blackest point identified by the subjective AVM and the iodine-unstained area. In cases without an iodine-unstained area, only the blackest point was included in the biopsy specimen. Conversely, in cases without the blackest point, only iodine-unstained area was included in the biopsy specimen. In cases with neither a blackest point identified by the AVM nor an iodine-unstained area, the area macroscopically suspected to be the lesion was resected as the biopsy specimen.

Histopathological diagnosis of SCC and leukoplakia with dysplasia (LwD) was defined as the epithelial dysplasia-positive group, and that of leukoplakia without dysplasia (Lw/oD) was defined as the epithelial dysplasia-negative group. The criteria of histopathological diagnosis of epithelial dysplasia were in accordance with WHO criteria^28^ (Table 2).

### Calculation of the luminance ratio

The photographs of the subjective AVM images viewed from the VELscope were analyzed by Image J software (ver 1.47; National Institutes of Health, Bethesda, MD, USA), and the luminance ratio was calculated. First, all photographs were converted from color images to grayscale, and the luminance level was measured by a precise circle with a 12-pixel diameter. The blackest point of FVL area was defined as the luminance level of the lesion, and the average luminance level of three points in the white area of the same image, which was originally green light area, was defined as the luminance level of the healthy area. The LNR was then determined using the following formula: LNR=luminance of the healthy area/luminance of the lesion.

### Setting of the objective AVM

**Figure 2:**
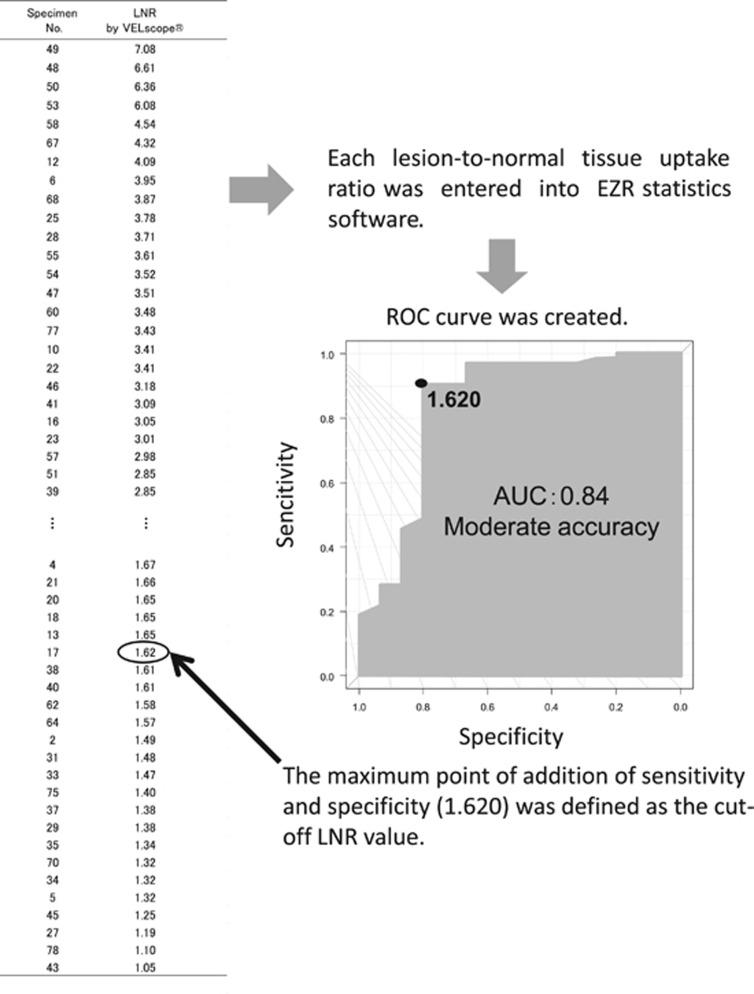
**Schema of definition of cutoff value.** First, each lesion-to-normal tissue uptake ratio was entered into EZR statistics software, and a receiver operating characteristic curve was created. The area under the curve was 0.84, indicating moderate accuracy. Next, the maximum point of addition of the sensitivity and specificity (1.620) was defined as the cutoff value of lesion-to-normal tissue uptake ratio.

First, the detection accuracy for epithelial dysplasia of the tongue using the subjective AVM and the IOM was calculated on the basis of the histopathological diagnosis. Next, a receiver operating characteristic (ROC) curve was created by EZR statistics software (Saitama Medical Center, Jichi Medical University, Saitama, Japan; http://www.jichi.ac.jp/saitama-sct/SaitamaHP.files/statmedEN.html, accessed 28 October 2015).^29^ The maximal point of addition of the sensitivity and specificity on the ROC curve was defined as the cutoff LNR value according to the Youden index,^30^ which is the criterion of determination of the existence of FVL (Figure 2). Patients’ list was then rearranged in order of the LNR. When the LNR was higher than the cutoff value, the lesion was considered FVL-positive and dysplasia-positive; when the LNR was lower than the cutoff value, the lesion was considered FVL-negative and dysplasia-negative, which were the criteria of the objective AVM. Finally, the detection accuracy for epithelial dysplasia by the objective AVM was also calculated with comparing to the histopathological diagnosis.

### Statistical analyses

To evaluate the potential clinical usefulness of the objective AVM, the luminance ratio was compared between the pathologically diagnosed epithelial dysplasia-positive and -negative groups. The kappa statistic was then calculated as the conformity ratio between the histopathological analysis and each auxiliary examination method (the subjective AVM, the IOM and the objective AVM), and the Mann–Whitney *U*-test was conducted for comparison within groups.

## Results

### Results of the subjective AVM and the IOM

The subjective AVM showed 66 (83.5%) FVL-positive and 13 (16.5%) -negative specimens. On the other hand, the conventional IOM showed 74 (93.7%) iodine-unstained areas and 5 (6.3%) iodine-stained areas (Table 3).

### Typical results of the subjective AVM, the conventional IOM and the histopathological diagnosis

**Figure 3:**
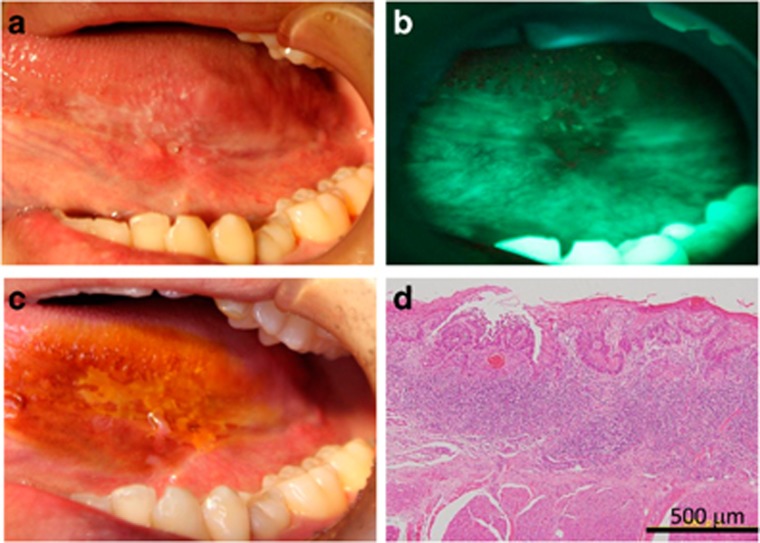
**Typical lesion well detected by both autofluorescence visualization method and iodine-staining method.** (**a**) Macroscopically, there was an outwardly growing lesion on the lateral edge of the tongue. The lesion showed (**b**) fluorescence visualization loss with fluorescent light emitted from the VELscope and (**c**) an iodine-unstained area by the iodine-staining method. (**d**) Histopathologically, a multi-layered epithelium and destroyed structure of the epithelial basal membrane were observed leading to diagnosis of squamous cell carcinoma. Bar=500 μm.

Patient No. 12 was one of the typical type of patients for whom the subjective AVM and the conventional IOM could detected the epithelial dysplasia lesion well. There was lace-like white plaque at the lateral edge of the tongue, which was macroscopically suspected to be epithelial dysplasia. With VELscope emission light, the area of suspected epithelial dysplasia was detected as black area (FVL) and surrounding healthy area turned bright white. The lesion was also detected as iodine-unstained area by the conventional IOM. Histopathologically, hematoxylin and eosin staining of the lesion showed destruction of the basement membrane and tumor cell invasion into the submucosal tissue with keratin pearl formation, resulting the diagnosis of oral SCC (Figure 3).

### Combinatorial results of histopathological diagnosis and the subjective AVM or IOM

The histopathological diagnosis of all 79 specimens was SCC including carcinoma *in situ* in 30 specimens, LwD in 34 specimens, and Lw/oD in 15 specimens. Therefore, 64 specimens were epithelial dysplasia-positive, and 15 were epithelial dysplasia-negative. Among the 64 histopathological epithelial dysplasia-positive specimens, the number of FVL-positive or -negative specimen was 55 or 9 in the subjective AVM, and that of iodine -unstained or -stained specimen was 61 or 3, respectively (Table 3). In contrast, among 15 pathological epithelial dysplasia-negative specimens, the number of FVL-positive or -negative specimen was 11 or 4 in the subjective AVM, and that of iodine -unstained or -stained specimen was 13 or 2, respectively.

### Lesion normalized ratio and the objective AVM

The average LNR of all specimens was 2.52 (range, 1.05–7.08). Based on the combinatorial results of the LNR and pathological diagnosis, an ROC curve was created. The area under the ROC curve was 0.84, which was moderately accurate, and the 95% confidence interval (CI) was 0.69–0.98. On the created ROC curve, the maximal point of addition of sensitivity and specificity was 1.620, which was defined as the cutoff value for the criterion of judgment of epithelial dysplasia for the objective FVL area (Figure 2). Thus, there were 61 FVL-positive specimens and 18 FVL-negative specimens examined by the objective AVM. Next, the combinatorial results of the objective AVM and histopathological diagnosis were analyzed. Among the 64 pathologically epithelial dysplasia-positive specimens, the number of FVL-positive or -negative specimen was 58 or 6 in the objective AVM, respectively; among the 15 epithelial dysplasia-negative specimens, 12 were FVL-negative (Table 3).

**Figure 4:**
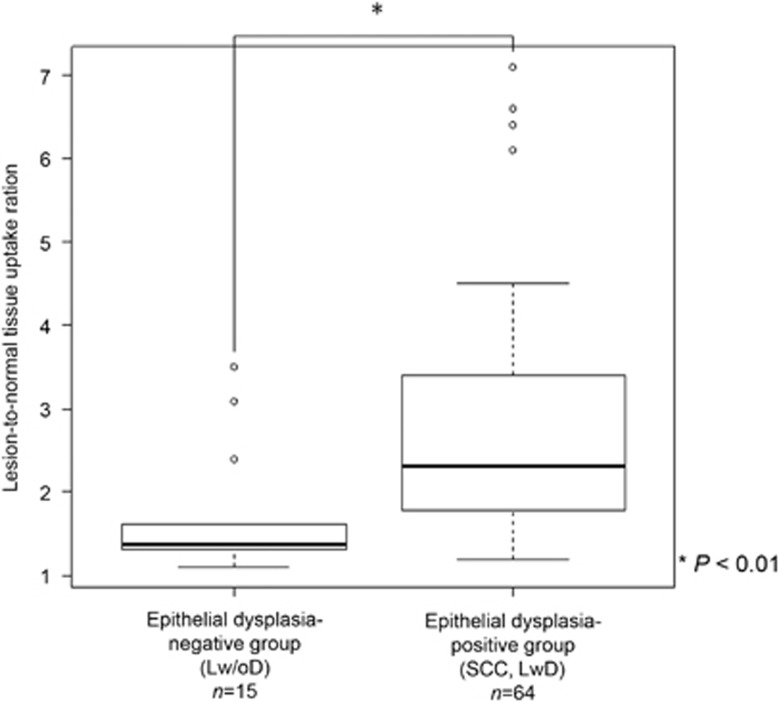
**Distribution of the lesion-to-normal tissue uptake ratio in the epithelial dysplasia-positive and -negative groups.** The lesion-to-normal tissue uptake ratio of the dysplasia-positive group was 2.71, which was significantly higher than that of the dysplasia-negative group (1.68) (*P*<0.000 1).

The average LNR in the histopathologically epithelial dysplasia-positive group was 2.71 (mean, 2.27; range, 1.25–7.08) and that in the epithelial dysplasia-negative group was 1.68 (mean, 1.38; range, 1.05–3.52). The LNR in the epithelial dysplasia-positive group was significantly higher than that in the epithelial dysplasia-negative group (*P*<0.000 1) (Figure 4).

### Correlation of the objective AVM and the conventional IOM

We also analyzed the correlation of the objective AVM and the conventional IOM by combining the result of the two methods. This analysis showed that 58 specimens (73.4%) were objectively FVL-positive and iodine-unstained, 3 specimens (3.8%) were FVL-positive without an iodine-unstained area, 16 specimens (20.3%) were FVL-negative and iodine-unstained, and 2 specimens (2.5%) were FVL-negative without an iodine-unstained area. All specimens with both objectively FVL-positive and iodine-unstained lesions were histopathologically diagnosed as epithelial dysplasia-positive.

The 12 of 15 (80.0%) specimens histopathologically diagnosed as epithelial dysplasia-negative were FVL-negative; however, 13 of these 15 (86.7%) specimens showed an iodine-unstained area, and only 2 (13.3%) showed an iodine-stained area (Table 3).

### Detection accuracy and degree of confidence of each modality

The detection accuracy of the subjective AVM, the IOM and the objective AVM for the diagnosis of epithelial dysplasia of the tongue was as follows: sensitivity; 85.9%, 95.3%, and 90.6% specificity, 26.7%, 13.3%, and 80.0% positive predictive value, 83.3%, 82.4%, and 95.1% negative predictive value, 30.8%, 40.0% and 66.7% accuracy, 74.7%, 79.7%, and 88.6% positive likelihood ratio, 1.17, 1.10, and infinity; and negative likelihood ratio, 0.53, 0.35, and 0.12, respectively. The degree of confidence between the histopathological diagnosis and the results of these auxiliary diagnostic modalities were as follows; the subjective AVM was fair with a kappa value of 0.132 (95% CI, −0.196–0.461), IOM was slight with a kappa value of 0.116 (95% CI, −0.271–0.503) and the objective AVM was substantial with a kappa value of 0.656 (95% CI, 0.444–0.868) (Table 3).

### False-negative and false-positive specimens

**Figure 5:**
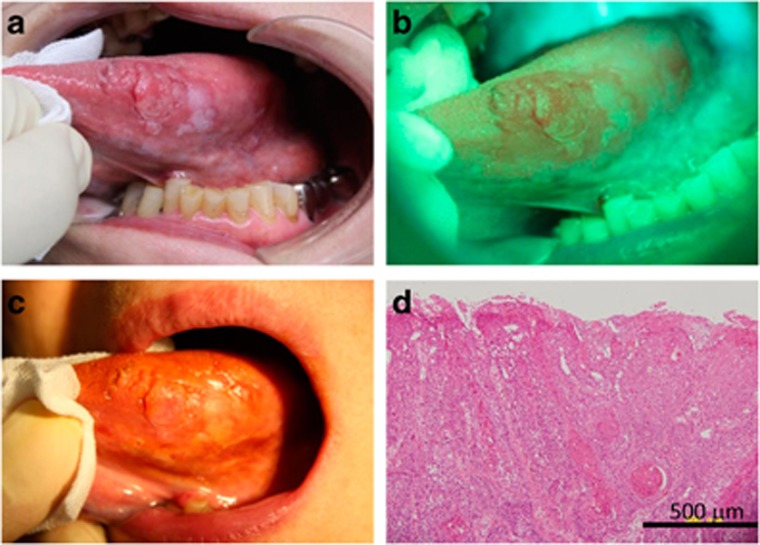
**False-negative specimen by the objective autofluorescence visualization method.** (**a**) There was an outwardly growing lesion on the lateral edge of the tongue. (**b**) The lesion did not show apparent fluorescence visualization loss with fluorescent light emitted from the VELscope leading to low luminance ratio. One of the reasons could be that the lesion is located on the lateral edge of the tongue. Another reason could be possible genetic alteration of the specimen. (**c**) However, the lesion was iodine-unstained. (**d**) The histopathological diagnosis was squamous cell carcinoma (Specimen No. 2). Bar=500 μm.

**Figure 6:**
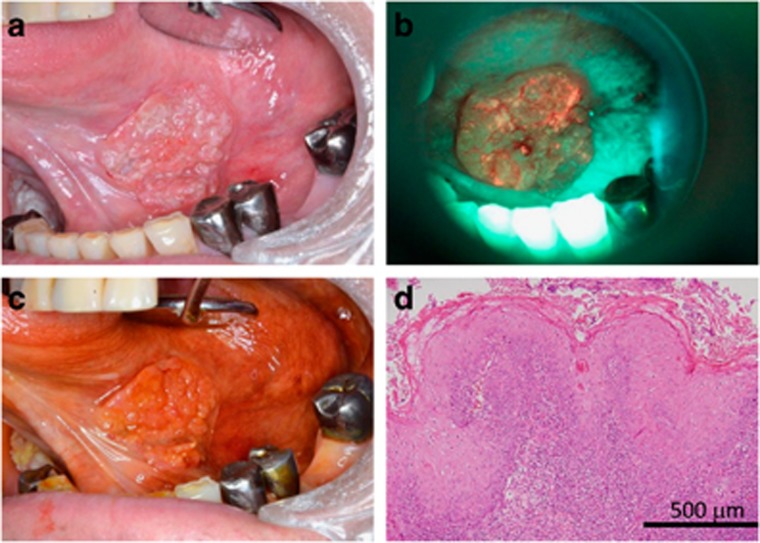
**False-negative specimen by the iodine-staining method.** (**a**) There was also an outwardly growing lesion on the lateral edge of the tongue. (**b**) The lesion showed relatively apparent fluorescence visualization loss with fluorescent light emitted from the VELscope. (**c**) The same lesion was iodine-unstained. (**d**) The histopathological diagnosis was squamous cell carcinoma (Specimen No. 4). Bar=500 μm.

Six specimens were assessed as false-negative by the objective AVM. Histopathologically, two were assessed as SCC and four were assessed as LwD. Specimen No. 2 was typical type of false-negative by the objective AVM. There was macroscopically suspected lesion area with iodine-unstained area, however, the FVL was not apparent, and the histopathological diagnosis was SCC (Figure 5). On the other hand, three specimens were assessed as false-negative by the conventional IOM. Histopathologically, one was diagnosed as SCC and two were diagnosed as LwDs. In contrast to the false-negative cases by the objective AVM, the macroscopically suspicious lesion areas were iodine-stained with relatively apparent FVL, and the histopathological diagnosis was also SCC (Figure 6). The false-negative specimen differed within the IOM and the objective AVM groups. Thirteen specimens were assessed as false-positive by the IOM, three specimens were assessed as false-positive by the objective AVM, and all of them were diagnosed as LwDs.

## Discussion

In this study, we tried to quantify the luminance of FVL for the objective AVM and calculated a cutoff value of 1.62. This is the first attempt to provide an AVM device capable of objectively evaluating the viewing function of an eye to be examined. The present study thus contains significant meaning for the diagnosis and treatment of oral cancer including epithelial dysplasia.

Epithelial dysplasia has been reported to transform to oral SCC,^31^ and a statistically significant difference has been found in the malignant transformation rate of patients with leukoplakia with epithelial dysplasia *vs* leukoplakia without epithelial dysplasia.^32^ Although leukoplakia with moderate or severe dysplasia would be selectively and immediately excised after biopsy, while that with mild dysplasia would not be so hastly treated, there is still a significant difference in the transformation rate of malignancy. However, there are opposite opinions regarding the relationship between malignant transformation and epithelial dysplasia.^31, 33, 34^ Because the statistically significant reported risk factors for malignant transformation of leukoplakia include the presence of epithelial dysplasia,^35^ we support the opinion that surgical resection with an adequate safety margin including the area of epithelial dysplasia would be effective in preventing malignant transformation.

The number of patient in this study might have been relatively low, and the number of pre-malignant and malignant specimens was higher than that of leukoplakia without dysplasia specimens. There are two plausible reasons. One is that the patients who were subjectively diagnosed as non-malignancy were emitted before examination by the two specialists. These patients had been diagnosed as lichen planus, pemphigus, and papilloma; they also received treatment and were undergoing careful follow-up at the time of this writing. The other plausible reason is that our hospital is one of two university-hospitals in Kanagawa prefecture that have an oral and maxillofacial surgery department; thus, our patients tend to have more severe diagnoses than patients in the oral and maxillofacial surgery departments of our general branch hospitals.

In the present study, the objective AVM showed higher specificity, positive predictive value, negative predictive value and accuracy than did the subjective AVM and the conventional IOM. In particular, high positive and negative predictive values can provide facilitate decision-making regarding the adequate safety margin at biopsy or excision surgery. In addition, the degree of confidence between the histopathological diagnosis and the objective AVM was higher than that of the other two modalities, indicating optimistic potential for this technique as auxiliary methods for diagnosis of epithelial dysplasia.

However, there are several concerns regarding the objective AVM; one is the slightly higher false-negative ratio compared with the conventional IOM. The correlation between the objective AVM and the conventional IOM was relatively high in epithelial dysplasia-positive patients, which suggests that both methods have a certain level of reliability. The most plausible reason for the slightly higher false-negative diagnosis of the objective AVM could be related to the location of the lesion and the healthy area. All false-negative lesions detected by the objective AVM were on the lateral edge of the tongue, and the healthy area was on the dorsum of the tongue.

The lesion showed relatively low FVL and the healthy area on the dorsome of tongue showed a relatively low level of green fluorescence, which led to a decreased luminance ratio between the lesion and healthy areas resulting a false-negative diagnosis (Figure 5). This hypothesis is also supported by Koch who reported that a lesion limited to the dorsum of the tongue would not be detected by the subjective AVM.^27^ Therefore, the setting of the AVM has to be reconsidered when the healthy area is located only on the dorsum of the tongue. Another plausible reason for the slightly higher false-negative diagnosis associated with the objective AVM is that the protocol of the objective AVM involves many steps to achieve a diagnosis, and a complicated calculation is required to determine the LNR and perform analysis using Image J software.

Interestingly, the false-negative patients of the objective AVM and the conventional IOM were totally different. The reason for this could be the difference in the mechanism to detect epithelial dysplasia. Moreover, we consider that the genetic alteration in each specimen might have differed, leading to the different level of FVL and patterns of false-negative results among the methods. Further investigation is necessary to evaluate the relationship between the false-negative pattern and the expression level of the reported molecular changes associated with dysplasia or transformation to oral SCC.^36^

In this study, only tongue lesions were analyzed to compare the objective AVM with the other two methods because adequate number of patients has not yet been analyzed. One of the advantages of both AVM techniques is that they are indicated for evaluation of keratinized mucosa, such as the gingiva and hard palate. We hypothesize that the cutoff value of each anatomic sites may be different, however, we do not yet know how these factors are related to endogenous fluorescence substances or what kind of genetic alteration which relates to the level of endogenous fluorescence substances will be found in epithelial dysplasia area and affect LNR. Therefore, further investigation for other sites of the oral cavity and reconsideration of the setting of the objective AVM would be required.

At this moment, chair-side evaluation by the objective AVM is available and the cutoff value for tongue lesion was decided in this report. For future feasibility of the objective AVM, cutoff value for another site of lesion in the oral cavity will be required at least. Then, software developing that minimize the time consumption will also be required.

Coincidently, Yan *et al.*^13^ had just reported a new attempt of objective methods of EVINCE by calculating ROC curve, area under the curve, and regions of interest, whose methods were similar to ours and their concept was probably based on same intensions to ours. Having said that, there are three different points from our study in addition to difference of devices as follows; (1) our study proposed comparison of three modalities (the subjective and objective AVM of VELscope and IOM); (2) our study also proposed comparison of LNR between different histopathological diagnosis; and (3) the first step of examination was performed just before biopsy, which is not chair-side usage of the device. Considering these two manuscripts, building objective and reliable methods for optical imaging system would be one of the next subjects to develop visual inspection devices.

When the objective AVM will be used in practice, it will be indicated not only for decision of biopsy or excision, but also observation after surgical treatment and screening for oral epithelial dysplasia at general clinics.

In conclusion, the objective AVM is suggested to have optimistic potential as a clinical auxiliary method comparable with the conventional IOM, but still may miss overt carcinomas.

## Figures and Tables

**Table 1:** Patient characteristics

Patient no.	Gender	Age	Specimen no.	Subjective AVM	IOM	Objective AVM		Histopathological diagnosis
						LNR	FVL	
1	F	65	1	−	+	1.77	+	Squamous cell carcinoma
			2	−	+	1.49	−	Squamous cell carcinoma
2	M	52	3	+	+	1.80	+	Squamous cell carcinoma
			4	+	+	1.67	+	Squamous cell carcinoma
3	M	70	5	−	+	1.32	−	Squamous cell carcinoma
4	M	75	6	+	−	3.95	+	Squamous cell carcinoma
5	F	74	7	+	+	1.92	+	Leukoplakia with mild to focal moderate dysplasia
			8	−	+	1.86	+	Leukoplakia with mild to focal moderate dysplasia
6	F	70	9	+	+	2.75	+	Squamous cell carcinoma
7	M	57	10	+	+	3.41	+	Leukoplakia with mild dysplasia
8	M	57	11	+	+	2.45	+	Leukoplakia with mild dysplasia
9	M	38	12	+	+	4.09	+	Squamous cell carcinoma
10	M	71	13	+	+	1.65	+	Leukoplakia with mild dysplasia
11	F	76	14	+	+	1.71	+	Leukoplakia with moderate dysplasia
			15	+	+	1.69	+	Leukoplakia with mild dysplasia
12	M	27	16	+	+	3.05	+	Squamous cell carcinoma
13	M	21	17	+	+	1.62	+	Carcinoma *in situ*
14	F	85	18	+	+	1.65	+	Epithelial dysplasia, moderate
15	M	65	19	+	+	1.81	+	Squamous cell carcinoma, invasive
16	M	37	20	+	+	1.65	+	Squamous cell carcinoma
17	M	61	21	−	+	1.66	+	Epithelial dysplasia, mild
18	F	26	22	+	+	3.41	+	Epithelial dysplasia, moderate to severe dysplasia
19	M	39	23	+	−	3.01	+	Leukoplakia with mild dysplasia
			24	+	−	1.98	+	Leukoplakia with mild dysplasia
20	F	65	25	+	+	3.78	+	Squamous cell carcinoma, invasive
21	M	56	26	+	+	2.79	+	Squamous cell carcinoma, invasive
			27	+	+	1.19	−	Hyperplastic epithelium
22	F	63	28	+	+	3.71	+	Squamous cell carcinoma, moderate differentiated
			29	+	+	1.38	−	Leukoplakia without dysplasia
23	F	64	30	+	+	1.88	+	Leukoplakia with mild dysplasia and lichenoid reaction
			31	−	+	1.48	−	Leukoplakia with mild dysplasia and lichenoid reaction
24	F	68	32	+	+	2.62	+	Leukoplakia with mild dysplasia
25	F	60	33	+	+	1.47	−	Leukoplakia without dysplasia
26	M	55	34	+	+	1.32	−	Leukoplakia without dysplasia
27	F	72	35	−	+	1.34	−	Leukoplakia without dysplasia
28	M	55	36	+	+	2.83	+	Squamous cell carcinoma
29	F	68	37	+	+	1.38	−	Leukoplakia without dysplasia
30	F	70	38	+	−	1.61	−	Leukoplakia without dysplasia
31	M	29	39	+	+	2.85	+	Squamous cell carcinoma
32	F	66	40	+	+	1.61	−	Leukoplakia without dysplasia
33	M	52	41	+	+	3.09	+	Leukoplakia without dysplasia
34	M	65	42	+	+	2.20	+	Leukoplakia with moderate dysplasia
35	M	55	43	−	−	1.05	−	Leukoplakia without dysplasia
36	F	44	44	+	+	1.79	+	Leukoplakia with mild to moderate dysplasia (oral epithelial dysplasia)
			45	−	+	1.25	−	Leukoplakia with mild to moderate dysplasia (oral epithelial dysplasia)
37	F	83	46	+	+	3.18	+	Oral epithelial dysplasia, moderate dysplasia compatible with leukoplakia with moderate dysplasia
38	F	81	47	+	+	3.51	+	Epithelial dysplaisa, moderate comoatible with leukoplakia with moderate dysplasia
			48	+	+	6.61	+	Epithelial dysplaisa, moderate comoatible with leukoplakia with moderate dysplasia
39	M	73	49	+	+	7.08	+	Epithelial dysplasia, moderate compatible with leukoplakia with moderate dysplasia
			50	+	+	6.36	+	Epithelial dysplasia, moderate compatible with leukoplakia with moderate dysplasia
40	F	67	51	+	+	2.85	+	Epithelial dysplasia, mild
			52	+	+	2.29	+	Squamous cell carcinoma
41	F	51	53	+	+	6.08	+	Squamous cell carcinoma
42	F	80	54	+	+	3.52	+	Epithelial hyperplasia and erosion
43	M	68	55	+	+	3.61	+	Squamous cell carcinoma
			56	+	+	1.81	+	epithelial dysplasia
			57	+	+	2.98	+	Squamous cell carcinoma
44	F	44	58	+	+	4.54	+	Squamous cell carcinoma
45	M	72	59	+	+	2.13	+	Epithelial dysplasia, mild leukopakia with mild dysplasia
46	M	77	60	+	+	3.48	+	Squamous cell carcinoma, well differentiated
47	F	29	61	+	+	1.96	+	Squamous cell carcinoma
48	F	85	62	+	+	1.58	−	Epithelial dysplasia, mild leukoplakia with mild dysplasia
			63	+	+	1.73	+	Epithelial dysplasia, mild leukoplakia with mild dysplasia
49	F	55	64	+	+	1.57	−	Epithalial dysplasia, mild leukoplakia with mild dysplasia
50	M	45	65	+	+	2.16	+	Epithelial dysplasia, mild leukoplakia with mild dysplasia
51	M	75	66	−	+	1.95	+	Leukoplakia with mild to moderate dysplasia and ulceration
52	F	55	67	+	+	4.32	+	Squamous cell carcinoma
			68	+	+	3.87	+	Squamous cell carcinoma
53	F	60	69	+	+	2.42	+	Squamous cell carcinoma
54	M	43	70	−	+	1.32	−	Epithelial hyperplasia, so called hyperparakeratosis
55	F	58	71	+	+	2.38	+	Squamous cell carcinoma, invasive
56	M	57	72	−	+	1.98	+	Oral epithelial dysplasia, mild (SIN1) leukoplakia with mild dysplasia
57	M	59	73	+	+	2.13	+	Oral epithelial dysplasia, moderate leukoplakia with moderate dysplasia
58	F	50	74	+	+	2.76	+	Squamous cell carcinoma
59	M	57	75	−	+	1.40	−	Epithelial hyperplasia, leukoplakia without dysplasia
			76	+	+	2.37	+	Epithelial hyperplasia, leukoplakia without dysplasia
60	F	77	77	+	+	3.43	+	Oral epithelial dysplasia, moderate leukoplakia with moderate dysplasia
61	F	69	78	+	+	1.10	−	Epithalial hyperplasia
62	M	54	79	+	+	2.26	+	Squamous cell carcinoma

AVM, autofuorescence visualization; F, female; FVL, fuorescence visualization loss; IOM, iodine-staining method; LNR, lesion-to-normal tissue uptake ratio; M, male.

Gender: 31 male, 31 female.

Age: average 59.6 years, median 60.5 years, range 21–85 years.

Site of the lesions: tongue.

**Table 2:** 

Architecture	Cytology
Irregular epithelial stratification	Abnormal variation in nuclear size (anisonucleosis)
Loss of polarity of basal cells	Abnormal variation in nuclear shape (nuclear pleomorphism)
Drop-shaped rate ridges	Abnormal variation in cell size (anisocytosis)
Increased number of mitotic figures	Abnormal variation in cell shape (cellular pleomorphism)
Abnormally superficial mitoses	Increased nuclear-cytoplasmic ratio
Premature keratinization in single cells (dyskeratosis)	Increased nuclear size
Keratin pearls within rate pegs	A typical mitotic figures
	Increased number and size of nucleoli hyperchromasia

**Table 3:** Comparison of histopathological diagnosis and detection accuracy between objective AVM and IOM

Items	Histopathological diagnosis epithelial dysplasia		Total	Kappa statistics (95% confident interval)
	Positive	Negative		
Subjective AVM				
FVL-positive	55	11	66	0.132
FVL-negative	9	4	13	(−0.196–0.461)
IOM				
Iodine-unstained area	61	13	74	0.116
Iodine-stained area	3	2	5	(−0.271–0.503)
Objective AVM				
FVL-positive	58	3	61	0.656
FVL-negative	6	12	18	(0.444–0.868)

	Sensitivity	Specificity	Positive predictive value	Negative predictive value	Accuracy	Positive likelihood ratio	Negative likelihood ratio
Subjective AVM	85.9%	26.7%	83.3%	30.8%	74.7%	1.17	0.53
IOM	95.3%	13.3%	82.4%	40.0%	79.7%	1.10	0.35
Objective AVM	90.6%	80.0%	95.1%	66.7%	88.6%	∞	0.12

AVM, autofluorescence visualization method; FVL, fluorescence visualization loss; IOM, iodine-staining method.
